# Coolant Wetting Simulation on Simplified Stator Coil Model by the Phase-Field Lattice Boltzmann Method

**DOI:** 10.3390/e24020219

**Published:** 2022-01-30

**Authors:** Makoto Sugimoto, Tatsuya Miyazaki, Masayuki Kaneda, Kazuhiko Suga

**Affiliations:** Department of Mechanical Engineering, Osaka Prefecture University, 1-1 Gakuen-cho, Naka-ku, Sakai 599-8531, Japan; sugimoto@htlab.me.osakafu-u.ac.jp (M.S.); miyazaki@htlab.me.osakafu-u.ac.jp (T.M.); suga@me.osakafu-u.ac.jp (K.S.)

**Keywords:** two-phase flow, square rod array, phase-field model, lattice Boltzmann method, oil cooling

## Abstract

Stator coils of automobiles in operation generate heat and are cooled by coolant poured from above. The flow characteristic of the coolant depends on the coil structure, flow condition, solid–fluid interaction, and fluid property, which has not been clarified due to its complexities. Since straight coils are aligned and layered with an angle at the coolant-touchdown region, the coil structure is simplified to a horizontal square rod array referring to an actual coil size. To obtain the flow and wetting characteristics, two-phase fluid flow simulations are conducted by using the phase-field lattice Boltzmann method. First, the flow onto the single-layered rod array is discussed. The wetting area is affected both by the rod gap and the wettability, which is normalized by the gap and the averaged boundary layer thickness. Then, the flow onto the multi-layered rod arrays is investigated with different rod gaps. The top layer wetting becomes longitudinal due to the reduction of the flow advection by the second layer. The wetting area jumps up at the second layer and increases proportionally to the below layers. These become remarkable at the narrow rod gap case, and finally, the dimensionless wetting area is discussed at each layer.

## 1. Introduction

**Figure 1:**
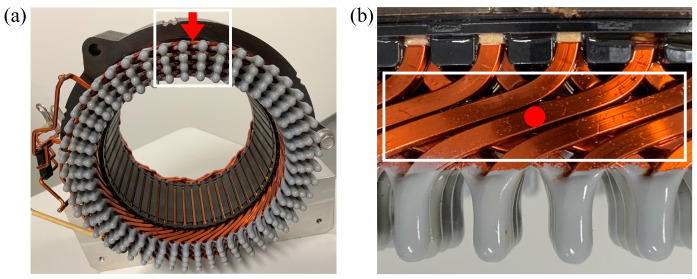
Stator coil of automobile with a coolant touchdown location: (**a**) Front view; (**b**) Top view.

Electrification of automobiles has been under the spotlight for reducing ${\mathrm{CO}}_{2}$ emissions. Among electric components in a vehicle, an electric motor is considered one of the most important ones for the powertrains, and its heat removal is crucial for overall improvement. The heat emitted from the electric motor consists of Joule losses, iron losses, stream load losses, and mechanical losses [1,2,3]. For heat removal from the vehicle motor, oil cooling has been used in terms of efficiency; the oil (electrically-insulated coolant) can directly touch the heat source and exchange the heat from the motor [4]. For example, the stator coil shown in Figure 1a, one of the main heat sources of the motor, is cooled by the oil poured from the nozzle holes above.

Ha et al. [5] investigated the cooling of a motor both experimentally and numerically. They reported that most of the oil flows through the stator coil at its crown part (uncovered part, Figure 1b) and the heat is exchanged therein. This suggests that the behavior of the oil at the uncovered coil area is crucial for heat removal efficiency. The oil behavior is affected by numerous parameters depending on the coil structure, pouring condition, the physical properties of the oil, etc. Multiple nozzle holes and/or oil inlets are employed to improve the flowing/wetting uniformity for the actual cooling system. Due to its complexity, flow characterization onto/into the structure has not been clarified yet.

At the uncovered coil region (white rectangle in Figure 1b), rectangular coils are aligned with a constant gap and layered with angles. This can be simplified to multi-layered horizontal rectangular rod arrays, which can reduce the parameters for the flow characterization. If the flowing condition is fixed, the representative parameters crucial for the wetting are structural (gap widths) and wettability (contact angles).

In terms of the research approach, visualization of the fluid on/inside the structure with various gap widths is difficult in experiments. Instead, a two-phase numerical simulation that can treat the complex structure can be an alternative method. Recently, the lattice Boltzmann method (LBM) has been attracting attention for use as an alternative computational fluid dynamics solver. The LBM offers exclusive features that include parallelization and computation on supercomputers and the ability to capture complex geometries [6]. This method has been extended to enable its use in multiphase flow solvers with various models such as the color-gradient model [7], the pseudopotential model (Shan–Chen model) [8], and the phase-field model [9]. The phase-field lattice Boltzmann method has been used in various applications such as the study of the effect of natural convection on lamellar eutectic growth [10] and the design of the hybrid phase change material-based metal hydride $H_{2}$ storage tank [11]. Our group has developed a phase-field lattice Boltzmann method with high phase conservation and precise wettability representation on complex geometries. This method was applied to the droplet infiltration into the porous media and the threshold of the wettability on the infiltration was found [12].

In this study, the characterization of the fluid flow and wetting is numerically investigated. A single-layer case is firstly investigated with the different wettabilities and rod gaps. Then, the cases for layered horizontal rod arrays are discussed with the rod gaps.

In this study, therefore, pouring liquid onto the multi-layered horizontal rectangular rod arrays is studied to clarify the characteristics. In particular, as a representative factor of the structure, the effect of the coil gap is investigated. It is expected that the fluid goes down faster at the wider rod gap. However, the relation between the fluid volume inside the structure and the gap, which is crucial for the cooling efficiency, has not been clarified nor characterized yet.

## 2. Numerical Schemes

In this study, the phase-field lattice Boltzmann method based on the conservative Allen–Cahn equation [12,13] is used for the two-phase flow simulation. The detailed numerical schemes are described as follows.

### 2.1. Governing Equations

In the present phase-field model, phases are distinguished using the order parameter ϕ defined as a local liquid volume fraction. Therefore, ϕ=0,1 denote the gas and liquid phases, respectively. The region where 0<ϕ<1 denotes a diffuse interface with finite thickness *W*. The time evolution of the order parameter ϕ is described by the following conservative Allen–Cahn equation,

(1) $$\frac{\partial \phi }{\partial t}+\nabla \cdot \left(\phi u\right)=\nabla \cdot \left[M_{\phi }\left(\nabla \phi -\lambda n\right)\right],$$

where $M_{\phi }$ is the mobility, $\lambda =4\phi \left(1-\phi \right)/W$, and n=∇ϕ/|∇ϕ| is the unit vector oriented normal to the liquid–gas interface.

The time evolution of the velocity u is described by the following Navier–Stokes equation,

(2) $$\frac{\partial \left(\rho u\right)}{\partial t}+\nabla \cdot \left(\rho uu\right)=-\nabla p+\nabla \cdot \left[\mu \left(\nabla u+\nabla u^{T}\right)\right]+F_{s}+F_{g},$$

where $F_{s}$ and $F_{g}$ are the following surface tension force term and the gravitational force term, respectively,

(3) $$F_{s}=\mu_{\phi }\nabla \phi ,\;\;F_{g}=\rho g,$$

where $\mu_{\phi }$ is the chemical potential, which is defined as [14]

(4) $$\mu_{\phi }=4\beta \phi \left(\phi -1\right)\left(\phi -\frac{1}{2}\right)-\kappa \nabla^{2}\phi ,$$

where β and κ are the physical parameters below, which are dependent on both the interfacial thickness *W* and the surface tension σ,

(5) $$\beta =\frac{12\sigma }{W},\;\;\kappa =\frac{3}{2}\sigma W.$$

### 2.2. Phase-Field Lattice Boltzmann Method

In this section, the governing equations are converted into lattice Boltzmann equations (LBEs). The conservative Allen–Cahn equation and the Navier–Stokes equation are converted into the following lattice Boltzmann equations,

(6) $$f_{i}\left(x+e_{i}\delta_{t},t+\delta_{t}\right)-f_{i}\left(x,t\right)=\Omega_{i}^{f}\left(x,t\right)+\delta_{t}R_{i}^{f}\left(x,t\right),$$

(7) $$g_{i}\left(x+e_{i}\delta_{t},t+\delta_{t}\right)-g_{i}\left(x,t\right)=\Omega_{i}^{g}\left(x,t\right)+\delta_{t}R_{i}^{g}\left(x,t\right),$$

where $f_{i}$ and $g_{i}$ are the distribution functions of the order parameter ϕ and the velocity u, respectively. $\Omega_{i}^{f}$ and $\Omega_{i}^{g}$ are the collision operators, $R_{i}^{f}$ is the source term, and $R_{i}^{g}$ is the force term. The subscript *i* is the discrete direction, $e_{i}$ is the discrete velocity, and $\delta_{t}$ is the time step. In this study, the single-relaxation-time (SRT) model [15] is used for $\Omega_{i}^{f}$. The weighted multiple-relaxation-time (WMRT) model [16] is used for $\Omega_{i}^{g}$ to improve the numerical stability. The collision operators are described as follows,

(8) $$\Omega_{i}^{f}=-\frac{1}{\tau_{f}}\left(f_{i}-f_{i}^{\mathrm{eq}}\right),$$

(9) $$\Omega^{g}=-M^{-1}S^{g}M\left(g-g^{\mathrm{eq}}\right),$$

where $f_{i}^{\mathrm{eq}}$ and $g_{i}^{\mathrm{eq}}$ are the local equilibrium distribution functions and M is the transformation matrix of the WMRT model. $S^{g}$ is the diagonal collision matrix given as follows,

(10) $$S^{g}=\mathrm{diag}\left(\underset{d+1}{\underset{︸}{1,1,1,1}},\underset{\left(d+2\right)\left(d-1\right)/2}{\underset{︸}{s_{g},s_{g},s_{g},s_{g},s_{g}}},\underset{q-d\left(d+3\right)/2}{\underset{︸}{1,1,...,1}}\right),$$

where $s_{g}=1/\tau_{g}$. *d* and *q* are the dimensions of the discrete velocity and the number of the discrete directions, respectively. The relaxation times $\tau_{f}$ and $\tau_{g}$ have the following relationships with the mobility $M_{\phi }$ and the kinematic viscosity ν, respectively,

(11) $$M_{\phi }=c_{s}^{2}\left(\tau_{f}-\frac{1}{2}\right)\delta_{t},$$

(12) $$\nu =c_{s}^{2}\left(\tau_{g}-\frac{1}{2}\right)\delta_{t},$$

where $c_{s}$ is the speed of sound.

The local equilibrium distribution function $f_{i}^{\mathrm{eq}}$ is obtained as follows using the first-order term of the Maxwell distribution

(13) $$f_{i}^{\mathrm{eq}}=w_{i}\phi \left(1+\frac{e_{i}\cdot u}{c_{s}^{2}}\right).$$

The local equilibrium distribution function $g_{i}^{\mathrm{eq}}$ is written as [13,17]

(14) $$g_{i}^{\mathrm{eq}}=\{\begin{array}{cc} \frac{p}{c_{s}^{2}}\left(w_{i}-1\right)+\rho s_{i}\left(u\right), & \left(i=0\right),\\ \frac{p}{c_{s}^{2}}w_{i}+\rho s_{i}\left(u\right), & \left(i\neq 0\right), \end{array}$$

where $w_{i}$ is the weight coefficient and i=0 is the discrete direction in which $e_{i}=0$. $s_{i}$ is the following function

(15) $$s_{i}\left(u\right)=w_{i}\left[\frac{e_{i}\cdot u}{c_{s}^{2}}+\frac{{\left(e_{i}\cdot u\right)}^{2}}{2c_{s}^{4}}-\frac{u\cdot u}{2c_{s}^{2}}\right].$$

The source term $R_{i}^{f}$ and the force term $R_{i}^{g}$ are required to recover the governing equations from the LBEs and are expressed as follows [16,18]

(16) $$R_{i}^{f}=\left(1-\frac{1}{2\tau_{f}}\right){\bar{R}}_{i}^{f},$$

(17) $$R^{g}=M^{-1}\left(I-\frac{S^{g}}{2}\right)M{\bar{R}}^{g},$$

where

(18) $${\bar{R}}_{i}^{f}=w_{i}e_{i}\cdot \left[\frac{\partial_{t}\left(\phi u\right)}{c_{s}^{2}}+\lambda n\right],$$

(19) $${\bar{R}}_{i}^{g}=w_{i}\frac{e_{i}\cdot \left(F_{s}+F_{g}\right)+\left(e_{i}\cdot u\right)\left(e_{i}\cdot \nabla \rho \right)}{c_{s}^{2}}.$$

In this study, the time derivative term in Equation (18) is computed using the following explicit Euler scheme

(20) $$\partial_{t}\left(\phi u\right)=\frac{\phi \left(t\right)u\left(t\right)-\phi \left(t-\delta_{t}\right)u\left(t-\delta_{t}\right)}{\delta_{t}}.$$

The macroscopic variables, comprising the order parameter ϕ, the velocity u, and the pressure *p*, are computed using the zeroth or first moments of the distribution functions as follows

(21) $$\phi =\sum_{i}f_{i},$$

(22) $$u=\frac{1}{\rho }\left[\sum_{i}e_{i}g_{i}+\frac{\delta_{t}}{2}\left(F_{s}+F_{g}\right)\right],$$

(23) $$p=\frac{c_{s}^{2}}{1-w_{0}}\left[\sum_{i\neq 0}g_{i}+\rho s_{0}\left(u\right)+\frac{\delta_{t}}{2}u\cdot \nabla \rho \right].$$

Moreover, the density ρ and the kinematic viscosity ν are interpolated using the order parameter as follows

(24) $$\rho =\phi \rho_{l}+\left(1-\phi \right)\rho_{g},$$

(25) $$\nu =\phi \nu_{l}+\left(1-\phi \right)\nu_{g},$$

where the subscripts l and g denote the physical properties of the liquid and gas phases, respectively.

In this study, the D3Q27 discrete velocity model [19] is used for both distribution functions.

## 3. Wetting Boundary Condition

**Figure 2:**
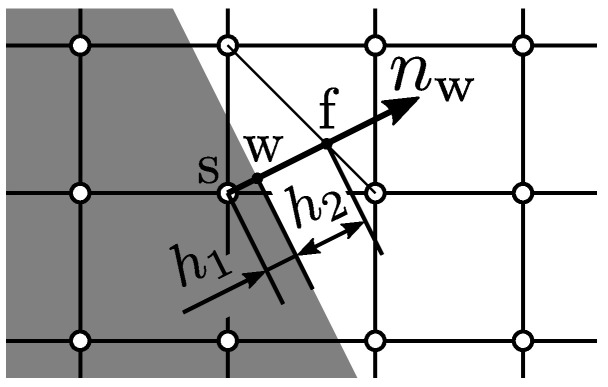
Schematic drawing of an example of wall and computational grid.

In this study, the level set function [20], which is a signed distance function from the wall, is used to represent the wall smoothly, and the cubic boundary condition (CBC) [12,21,22], which considers up to the third-order term of the wall free energy, is used to reproduce wetting. Figure 2 shows an example of the positional relationship between the wall surface and the grid points around it. For the sake of simplicity, a two-dimensional system is shown here, but the same applies to a three-dimensional system.

The CBC reproduces wetting by setting the contact angle θ as a computational parameter and imposing the order parameter gradient in the direction normal to the wall $\partial \phi /\partial n_{w}$ that satisfies the given contact angle as a Neumann boundary condition. Specifically, a virtual order parameter ${\left.\phi \right|}_{s}$ considering the wetting is extrapolated using the order parameter ${\left.\phi \right|}_{f}$ and the order parameter gradient ${\left.\partial \phi /\partial n_{w}\right|}_{w}$. The wall normal gradient of the order parameter is determined by the CBC as follows [22]:

(26) $${\left.\frac{\partial \phi }{\partial n_{w}}\right|}_{w}=-\sqrt{\frac{2\beta }{\kappa }}{\left.\phi \right|}_{w}\left(1-{\left.\phi \right|}_{w}\right)\cos\theta .$$

The virtual order parameter at the solid phase is extrapolated by the following equation [22]:

(27) $${\left.\phi \right|}_{s}=\frac{h_{1}+h_{2}}{2ah_{2}}\left[1+a-\sqrt{{\left(1+a\right)}^{2}-4a{\left.\phi \right|}_{f}}\right]-\frac{h_{1}}{h_{2}}{\left.\phi \right|}_{f},$$

where

(28) $$a=-h_{2}\sqrt{\frac{2\beta }{\kappa }}\cos\theta \neq 0,\;\;\left(\theta \neq 90^{\circ }\right).$$

Note that ${\left.\phi \right|}_{s}={\left.\phi \right|}_{f}$ in the case of $\theta =90^{\circ }$. The wetting is taken into account via the surface tension force term in Equation (3) by calculating ∇ϕ and $\nabla^{2}\phi$ using the finite difference method with ${\left.\phi \right|}_{s}$.

## 4. Model Structure and Computational Conditions

**Figure 3:**
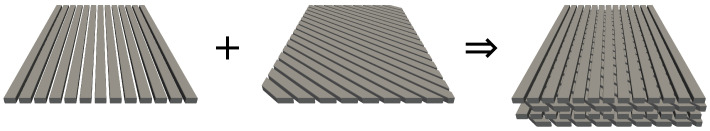
Stacking process of the multi-layered model.

The fluid flow and wetting are affected by the structure-dependent parameter (nozzle diameter and height to the layer, cross-section of the rod, rod width, and gap width), fluid dependent parameter (viscosity, surface tension), solid–fluid interaction parameter (wettability), and flow condition (flow rate), etc. In this study, the actual stator coil is modeled with the layered horizontal rod arrays. Each layer consists of an array of equally spaced and parallel aligned rods. The rod array layers are crossed with an angle of 45 degrees as shown in Figure 3. The cross-sectional shape of the rod is referred to the actual stator coil ($L_{v}/L_{h}=0.603$ and $L_{R}/L_{h}=8.45\times 10^{-2}$, where $L_{v}$ is the vertical length, $L_{h}$ is the horizontal length, and $L_{R}$ is the round chamfer radius).

**Figure 4:**
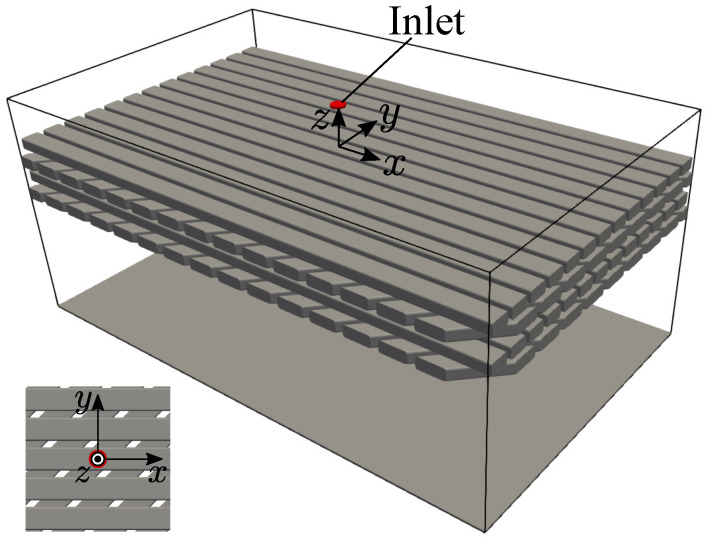
Computational domain of the stator coil modeled by horizontal square rod array.

The computational domain of the stator coil modeled by a horizontal square rod array is shown in Figure 4. The pouring nozzle has a round shape with a diameter $D=0.789L_{h}$, and it is located just above the rod. The height from the rod surface to the nozzle is 2.8D. In this study, the periodic condition is used at the *x* and *y* direction boundaries. A wall is placed at the −z direction boundary, and the outflow condition with the volume equal to the inflow volume is imposed on the +z direction boundary except for the nozzle in order to maintain the volume conservation of the entire system. On the solid surface, the no-slip condition is imposed by the interpolated bounce-back scheme [23], and the arbitrary wettability is imposed by the CBC [12,21,22].

The falling liquid is referred to the automatic transmission fluid of the automobile, which is used for electric vehicles, and the ambient gas is the air. The density ratio and the viscosity ratio of the liquid and gas are $\rho_{l}/\rho_{g}=705$ and $\mu_{l}/\mu_{g}=2.36\times 10^{3}$, respectively. The Bond number is $Bo=\left(\rho_{l}-\rho_{g}\right)\left|g\right|D^{2}/\sigma =1.81$, the Weber number is $We=\rho_{l}DU^{2}/\sigma =121$, and the Reynolds number is $Re=UD/\nu_{l}=74.9$, where *U* denotes the inlet velocity. In all simulations, the cross section of the square rods are resolved with $35.5\left(y\right)\times 21.4\left(z\right)$ grid points. The number of grid points for the entire system are different for each simulation because the liquid spreadings are different. The Cahn number, which represents the ratio of the interfacial thickness *W* to the characteristic length, is set to Ch=W/D=0.143 (*W* is resolved with 4 grid points). The other parameters are detailed in each section.

## 5. Validation

**Figure 5:**
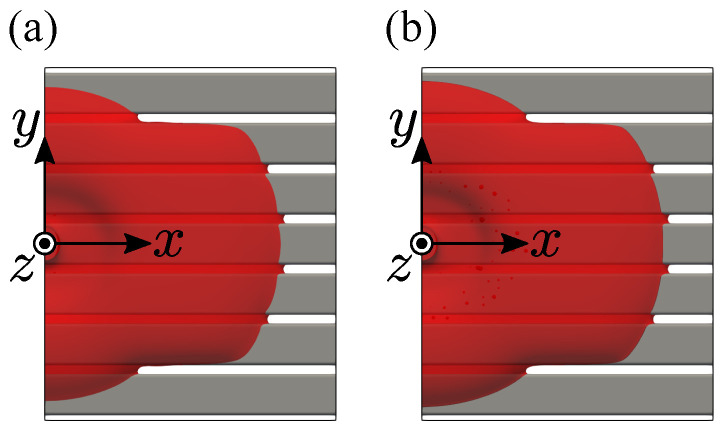
Top view of the wetting of single-layer at $L_{\mathrm{gap}}/L_{h}=0.282$, $\theta =30^{\circ }$, and tU/D=150: (**a**) Lower-resolution; (**b**) Higher-resolution.

Initially, to validate the number of grid points described in the previous section, the fluid flow simulation is carried out for the single-layer model. In this section, the dimensionless gap width is $L_{\mathrm{gap}}/L_{h}=0.211$, and the contact angle is 30 degrees. The simulations are performed for the conditions shown in the previous section and for the conditions in which the grid width is halved. Note that the interfacial thickness *W* is resolved with 4 grid points in both simulations. Figure 5 shows the top view at the dimensionless time tU/D=150. It is confirmed that the shape of the liquid is the same in these simulations. The wetting area obtained by the simulation with the number of grid points shown in the previous section is found to have a relative error of 4.24% compared to the higher-resolution simulation. Therefore, the number of grid points shown in the previous section is confirmed to be acceptable.

## 6. Results and Discussion

### 6.1. Wetting on Single-Layer—Effect of the Rod Gap and Wettability

Prior to the layered model, the fluid flow simulation is carried out for the single-layer model. This model consists of only the top layer of the layered one. In this section, the steady-state wetting area is discussed with different wettability (contact angle) and rod gap width. The contact angles simulated are 30 and 90 degrees. The dimensionless gap width $L_{\mathrm{gap}}/L_{h}$ are 0.282 and 0.423. For reference, a single rod case is also computed as the case of $L_{\mathrm{gap}}/L_{h}=\infty$.

**Figure 6:**
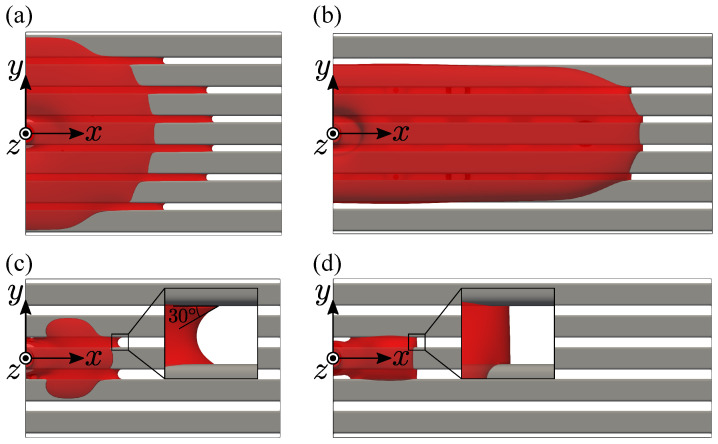
Top view of the steady-state wetting of single-layer: (**a**) $\theta =30^{\circ }$ and $L_{\mathrm{gap}}/L_{h}=0.282$; (**b**) $\theta =90^{\circ }$ and $L_{\mathrm{gap}}/L_{h}=0.282$; (**c**) $\theta =30^{\circ }$ and $L_{\mathrm{gap}}/L_{h}=0.423$; (**d**) $\theta =90^{\circ }$ and $L_{\mathrm{gap}}/L_{h}=0.423$.

Figure 6 shows the top view. In the narrower gap case ($L_{\mathrm{gap}}/L_{h}=0.282$), wetting depends on the rod’s wettability. The lower contact angle (30 degrees) induces the spanwise wetting with long menisci. Since the meniscus length depends on the contact angle, the lower contact angle induces the longer meniscus as long as the liquid bridges at the gap. By the long meniscus at the gap, the liquid can spread quasi-concentrically on the rod array. In contrast, at the higher contact angle case (90 degrees), the wetting becomes longitudinally with short menisci. This is because the short menisci suppress the liquid flow to the next rod, thus the longitudinal liquid transfer becomes dominant.

When the gap becomes wider ($L_{\mathrm{gap}}/L_{h}=0.423$), the wetting number of rods decreases to the center rod. The meniscus length becomes short regardless of the rod wettability and the longitudinal wetting of the center rod is almost the same. This is because the meniscus can not form between the wide gap due to the falling liquid by gravity, and the longitudinal wetting is restricted by the liquid at the gap.

**Figure 7:**
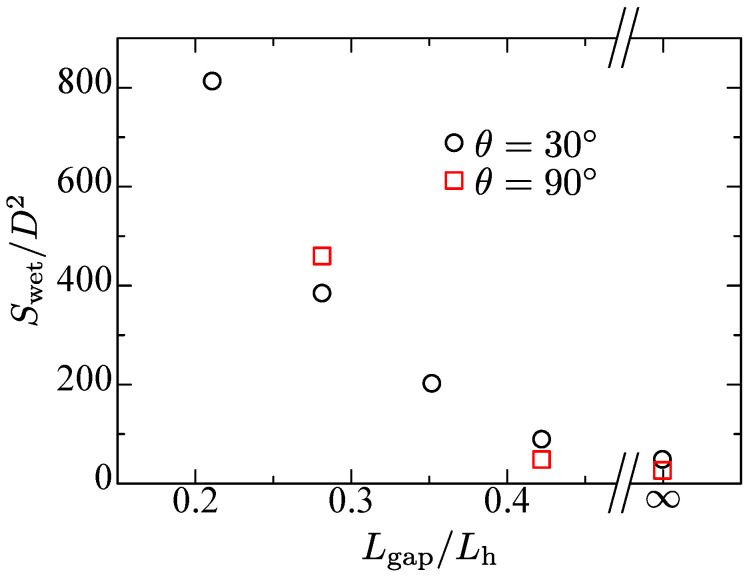
Dimensionless wetting area versus rod gap for single-layer with different contact angles.

Next, the wetting area is discussed. Figure 7 shows the dimensionless wetting area. The main dominant factor on the wetting area is the rod gap $L_{\mathrm{gap}}$. The abscissa is determined by the dimensionless rod gap $L_{\mathrm{gap}}/L_{h}$. The case of $L_{\mathrm{gap}}/L_{h}=\infty$ is also added in the figure.

It is found that the wetting area decreases proportionally to the threshold value $L_{\mathrm{gap}}/L_{h}\approx 0.4$ regardless of the contact angle and the wetting pattern. At the threshold rod gap, the number of wetting rods converges to 1 with the liquid between the rods. The wetting area is almost the same as the single rod case. The wetting area is largely affected by the rod gap, rather than that by the rod wettability.

### 6.2. Fluid Infiltration into the Layered Structure

**Figure 8:**
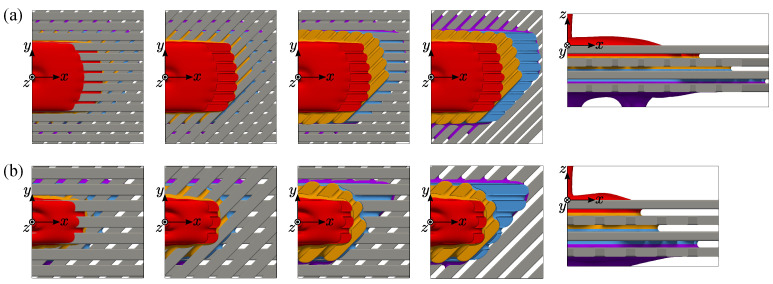
Top view of the steady-state wetting area at each layer: (**a**) $L_{\mathrm{gap}}/L_{h}=0.282$; (**b**) $L_{\mathrm{gap}}/L_{h}=0.423$.

The liquid wetting into the layered structure is discussed. In this section, the effect of the rod gap width is investigated at the contact angle of 30 degrees since the actual stator coil is lyophilic. Figure 8 shows the steady-state wetting at each layer. The liquid spreads symmetrically on the top array layer, and then that at the gaps falls to the second layer. On the second layer, the liquid spreads longitudinally. The menisci are formed not only between the horizontal rods but also between the lower side of the top layer and the upper side of the second layer. Since the rod array layers are crossed with an angle of 45 degrees, the overall wetting pattern has point symmetry. At each layer, the longitudinal wetting is enhanced more than the single-layer case because the menisci can be additionally formed between layers. These processes are repeated between the second and third layers and the third and fourth ones.

Comparing the top views of different gap widths, the wetting area is generally large for the narrower rod gap, which is the same tendency of the single-layer case. On the other hand, at the top layer, the longitudinal wetting becomes remarkable compared with the single-layer case (Figure 6a). As seen in the figure, the second layer supports the fluid at the bottom side of the top layer, including the meniscus area. The longitudinal wetting area at the reverse side decides the crossing wetting area at the lower layer, thus the wetting area at the lower layer, is enhanced, which is repeated as the fluid goes down.

### 6.3. Transient Wetting Area of the Layered Structure

**Figure 9:**
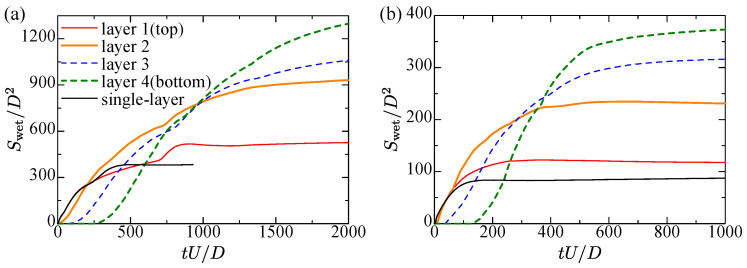
Transient wetting area at each layer: (**a**) $L_{\mathrm{gap}}/L_{h}=0.282$; (**b**) $L_{\mathrm{gap}}/L_{h}=0.423$.

To investigate the wetting in detail, the transient wetting area at each layer is shown in Figure 9. The elapsed time is non-dimensionalized by the nozzle diameter and the inlet velocity. The wetting area is normalized by the nozzle diameter. For reference, the case of the single-layer is added therein.

In both gap cases, the wetting on the top layer proceeds at the same pace as the single-layer case at the initial stage. A bit later, the onset of wetting on the second layer occurs before the wetting of the top layer finishes. Since the liquid on the second layer blocks the liquid from falling, the liquid on the top layer continues to spread longitudinally and then attains the constant value. For the second and lower layers, the wetting area becomes much larger than the top layer. This is because the liquid is supplied from the gap of the above layer, which is different from the top layer case from the single nozzle hole.

### 6.4. Steady-State Wetting Area in Layered Case

**Figure 10:**
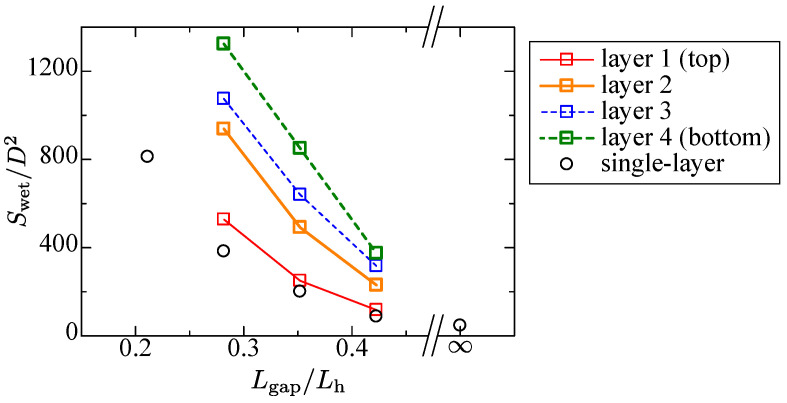
Steady-state wetting area at each layer.

The wetting phenomena attain the steady-state except the falling down liquid below the lowest layer. As shown in the prior section, the single-layer case suggests the wetting area is affected mainly by the rod gap. To confirm the tendency for the layered structure, the steady-state wetting area is arranged by the same scheme. Figure 10 shows the plot. The abscissa is composed of $L_{\mathrm{gap}}/L_{h}$. In the figure, the single-layer cases at various rod gaps are added for reference.

The wetting area of the top layer for the narrower gap case becomes a bit larger than the single-layer case, which corresponds to the top views in Figure 6a and Figure 8a. This is because the longitudinal wetting is enhanced by the second layer, whereas the wider rod gap case has almost the same wetting area as the single-layer case despite the different wetting patterns (Figure 6c and Figure 8b). In this case, the meniscus does not grow enough; thus, the longitudinal wetting is suppressed. Although the wetting area at the top surface is different from the single-layer case, it is interesting that the wetting area can be regarded to have the same tendency as the single-layer case with the dimensionless value employed herein.

For the lower layers, as also shown in Figure 8, the wetting area becomes larger at the narrower rod gap case, whereas in the wider gap case, the wetting area converges to the small range. This suggests that the permeability of the structure is crucial for wetting in the lower layer.

## 7. Conclusions

In this study, the wetting phenomena on the horizontal rod array are numerically investigated. The computed results for the single-layer show that the wetting area is largely affected by the rod gap and the wetting pattern by the rod wettability. When the rod array is layered, the top layer wetting becomes longitudinal in the narrower rod gap case, and the wetting area increases. The wetting area at the second layer jumps up compared with the top layer, which is due to the different inflow above the layer. Since each layer has longitudinal wetting, the wetting area in the transverse direction is dominant for the wetting area of the lower layer. For the wider rod gap case, these phenomena become less remarkable since the permeability of the structure becomes high. The wetting area can be normalized by the dimensionless parameter by the Reynolds number and dimensionless rod gap, in which the wetting at the top layer becomes similar to the single-layer case. These findings can contribute to the coil design of the electric motor to enhance the wetting and coolant exchange. In future work, we aim to apply the tendency obtained in this study to a wider range of conditions. We will also investigate the effect of the capillary number and other factors to develop a model that can predict the wetting with higher accuracy. We also plan to focus on the transient liquid infiltration phenomena. For this purpose, the approach using the Lucas–Washburn law [24,25] is considered to be effective. Additionally, we aim to implement a heat transfer solver using the LBM into the present method to further contribute to the cooling of stator coils.

## Data Availability

The data presented in this study are available on request from the corresponding author.

## Abbreviations

The following abbreviations are used in this manuscript:

CBC	Cubic Boundary Condition
LBE	Lattice Boltzmann Equation
LBM	Lattice Boltzmann Method
SRT	Single-Relaxation-Time
WMRT	Weighted Multiple-Relaxation-Time
